# Transcriptional profiling reveals upregulation of p53 signaling in porcine embryos produced in vitro^†^

**DOI:** 10.1093/biolre/ioaf113

**Published:** 2025-05-14

**Authors:** Rylie S Noland, Bethany K Redel, Marissa G LaMartina, Randall S Prather, Paula R Chen

**Affiliations:** Division of Animal Sciences, University of Missouri, Columbia, Missouri, USA; United States Department of Agriculture-Agricultural Research Service, Plant Genetics Research Unit, Columbia, Missouri, USA; Division of Animal Sciences, University of Missouri, Columbia, Missouri, USA; Division of Animal Sciences, University of Missouri, Columbia, Missouri, USA; United States Department of Agriculture-Agricultural Research Service, Plant Genetics Research Unit, Columbia, Missouri, USA

**Keywords:** porcine, embryo, in vitro, RNA-sequencing, p53, pifithrin, inhibitor

## Abstract

Although advances in the porcine embryo culture system have been achieved, the artificial environment continues to be stressful for the embryos, which hinders development. To identify areas of improvement, transcriptional profiling was performed on in vivo-derived, in vivo-matured and in vitro-cultured, and in vitro-matured and cultured porcine blastocyst-stage embryos. Numerous differentially expressed genes were detected between in vitro-cultured vs in vivo-derived (489 downregulated, 701 upregulated), in vitro-matured and cultured vs in vivo-derived (435 downregulated, 1124 upregulated), and in vitro-matured and cultured vs in vitro-cultured (32 downregulated, 168 upregulated). Moreover, Kyoto Encyclopedia of Genes and Genomes (KEGG) pathway analysis revealed upregulation of the p53 signaling pathway in vitro-matured and cultured embryos compared to in vivo-derived embryos. Therefore, in vitro-matured and cultured embryos were cultured with different p53 inhibitors, pifithrin-alpha, pifithrin-beta, or pifithrin-mu, to determine if the stress response could be suppressed to improve development to the blastocyst stage. Culture with 50 μM pifithrin-alpha improved development to the blastocyst stage (*P* < 0.05), but total cell number and transcript abundance of p53 target genes remained unaltered. No difference in development was observed after culturing embryos with pifithrin-beta, and embryos cultured with pifithrin-mu demonstrated decreased development. Lastly, two embryo transfers of embryos cultured with pifithrin-alpha demonstrated that the inhibitor did not disrupt developmental competence. Overall, the addition of pifithrin-alpha in the porcine embryo culture medium was shown to have beneficial effects on development and is suitable for generating live pigs with this system.

## Teaser

RNA-sequencing reveals that p53 signaling is upregulated in porcine embryos produced in vitro; addition of a p53 inhibitor, pifithrin-α, increased development to the blastocyst stage and produced developmentally competent piglets.

## Graphical Abstract

**Graphical Abstract:**
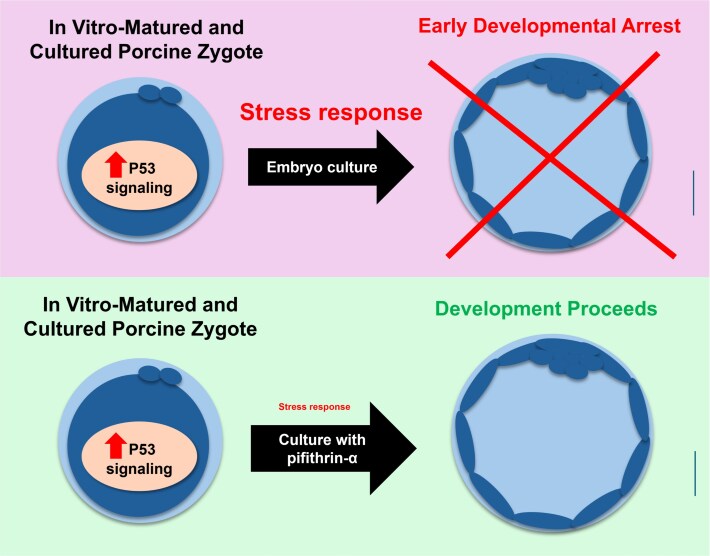

## Introduction

The use of pigs as models for agricultural and biomedical research is becoming increasingly popular; however, the in vitro production system is still generally inefficient. Before transfer into a surrogate, porcine embryos are exposed to an artificial environment during the first phase of development, which induces a stress response. As the demand for porcine models of human disease and agriculture grows, there is a great interest in improving the conditions of embryo culture and the efficiency of the system.

Transcriptional profiling is a powerful tool to pinpoint the requirements of preimplantation embryos in culture. By comparing the transcriptional profiles between in vivo*-*derived (IVV) embryos and in vitro-produced embryos, the discovery of differentially expressed transcripts related to amino acid transport and metabolism has led to experiments to correct this aberrant gene expression [1]. For example, an arginine transporter transcript was found to be upregulated in embryos produced in vitro*;*when arginine was added to the culture media, percentage of blastocyst stage embryos and other developmental parameters improved [2]. Subsequently, glutamine transport and metabolism transcripts were shown to be upregulated, and development to the blastocyst stage and mitochondrial function increased with addition of glutamine to the culture medium [3]. Moreover, the removal of a potentially unnecessary component in low oxygen culture—hypotaurine—was shown to have no detrimental effect on development and decreased the cost of producing the medium [4]. These alterations were ultimately given the name, MU4, for the fourth alteration to porcine zygote medium made by researchers at the University of Missouri (MU).

After making these improvements and 10 years after the original RNA-sequencing effort, it became necessary to perform transcriptional profiling once again on embryos cultured in this newest media formulation. With the current sequencing endeavor, several pathways were revealed to be dysregulated in the in vitro-produced embryos compared to IVV embryos; however, the p53 signaling pathway was shown to be upregulated in the entirely in vitro-produced embryos, indicating increased levels of stress. Inhibition of p53 signaling by using small molecules, called pifithrins, has been achieved in several cell types; however, the exact mechanism of action is still unknown [5, 6]. To overcome the stress response during in vitro culture, addition of various pifithrins to the medium was investigated for effects on development of the porcine embryos.

## Materials and methods

### Ethics statement

Collection of sow ovaries and use of live animals were in accordance with an approved protocol and standard operating procedures by the Animal Care and Use Committee of the University of Missouri—protocol number 28502.

### Chemical components

All compounds were obtained from Sigma Chemical Company unless otherwise stated.

### Embryo collection and culture for RNA-sequencing

Following artificial insemination of gilts, an oviduct and tip of the uterine horn were flushed on Day 2 to recover 4-cell stage embryos; these were cultured for 4 days in MU4, generating in vivo-matured and in vitro-cultured (IVC) blastocyst stage embryos. On Day 6, the gilts were euthanized, and the contralateral horns were flushed to obtain IVV blastocyst stage embryos. The third group of blastocyst stage embryos, referred to as in vitro-matured and cultured (IVMC), were created by aspirating cumulus-oocyte complexes from slaughterhouse-derived gilt ovaries, maturing and fertilizing in vitro, and culturing for 6 days in MU4. In all experiments, the incubator was maintained at 38.5°C with 5% CO_2_, 5% O_2_, and 90% N_2._

### RNA-sequencing and Kyoto Encyclopedia of Genes and Genomes pathway analysis

Total RNA was extracted from pools of 10 blastocyst stage embryos by using the RNeasy Micro Kit (Qiagen, Germantown, MD). The first and second strand cDNA was synthesized by the Genomics Technology Core at the University of Missouri. Three biological replicates per group were sequenced with the NovaSeq 6000 system (Illumina, San Diego, CA). Adapters, reads mapped to porcine rRNA genes, and reads mapped to the PhiX genome were removed before the remaining reads were mapped to *Sus scrofa*11.1 genome by using the default options in STAR (version 2.7.1a). Differential gene expression was tested by performing pairwise comparisons on the Bioconductor package DEseq2. Differentially abundant transcripts were detected between all groups of embryos with a false discovery rate (FDR) < 0.05 and log2 fold change ≥1. Venn diagrams to show overlap of comparisons were created with Venny 2.1. Differentially abundant transcripts were subjected to Kyoto Encyclopedia of Genes and Genomes (KEGG) pathway analysis by using gProfiler [7].

### Production of porcine embryos for culture with p53 inhibitors

Abattoir-derived ovaries were aspirated by using an 18-gage needle attached to a 10 mL syringe to obtain cumulus oocyte complexes. Those with at least one layer of cumulus cells and uniform cytoplasm were selected and matured in accordance with previous studies [3, 4]. The cumulus cells were removed from matured oocytes by vortexing in 0.03% hyaluronidase. Metaphase II oocytes with extrusion of the first polar body were washed, pooled in groups of 30–50, and placed in 50 μL droplets of modified Tris-buffered medium with a mineral oil overlay for fertilization. Spermatozoa were obtained from a single Landrace boar sample for each treatment replicate. Gametes were incubated together at 38.5°C in 5% CO_2_for 4 h before the transfer to MU4 embryo culture medium with or without pifithrins.

Pifithrins are commonly used in cell culture at a wide range of concentrations [5, 6, 8]; therefore, treated embryos were cultured with one of three different compounds, pifithrin-alpha (PFT-α; APExBio Technology, Houston, TX), pifithrin-beta (PFT-β; Selleck Chemicals, Houston, TX), or pifithrin-mu (PFT-μ; Stemcell Technologies, Vancouver, BC) by using a dose curve. Pifithrins were prepared by dissolving in dimethyl sulfoxide (DMSO). The same amount of DMSO was added to untreated groups as a vehicle control. Embryos were cultured at 38.5°C in a humidified atmosphere of 5% CO_2_, 5% O_2_, and 90% N_2_until Day 6 post-fertilization. To assess the effects of the p53 inhibitors, development to the blastocyst stage and total cell numbers were measured. Embryos were fixed in 2% paraformaldehyde at room temperature for 15 min and were stained with Hoechst 33342 for 15 min at 10 μg/mL to visualize nuclei.

### Terminal deoxynucleotidyl transferase dUTP nick end labeling assay

Day 6 blastocyst stage embryos from each group were fixed with 2% paraformaldehyde for 15 minutes and permeabilized with 0.1% Triton X-100 for 1 h at room temperature. Nuclei were labeled via incubation in 25 μL of terminal deoxynucleotidyl transferase dUTP nick end labeling (TUNEL) solution and label solution (fluorescein-conjugated dUTP and terminal dUTP; ThermoFisher, Waltham, MA) at 38°C for 30 min; nuclei with DNA damage will take up both solutions. Negative controls were incubated with the label solution only. Positive controls were incubated with 16 Kunitz units of DNase 1 in 25 μL of TUNEL solution to induce cleavage of double-stranded DNA. Blastocysts were washed three times in TL-HEPES, and nuclei were stained with 10 μg/mL Hoechst 33342 for 15 min. The stained blastocysts were mounted on slides and imaged with epifluorescence illumination with fluorescein isothiocyanate (FITC) and ultraviolet (UV) channels. The percentage of TUNEL-positive nuclei was determined out of the total number of nuclei and referred to as the apoptotic index.

### RNA extraction and complementary DNA synthesis

Day 6 blastocyst stage embryos cultured in 0 μM (control) or 50 μM PFT-α were pooled in groups of 10 for RNA isolation. The PicoPure™ RNA isolation kit (ThermoFisher) was used to extract total RNA, and the SuperScript™ IV VILO™ Master Mix (ThermoFisher) was used to generate cDNA. Each cDNA sample was used as a template for quantitative polymerase chain reaction (qPCR). Primers were designed by using Integrated DNA Technology software and are listed in Table 1(Idtdna.com; Coralville, IA, USA).

**Table 1:** Transcripts selected for quantitative PCR.

**Gene**	**Forward primer**	**Reverse primer**	**Accession number**
*BAX*	5′- CTCTGAGCAGATCATGAAGAC	5′- CTCGCTCAACTTCTTGGTAG	NM_001123183.1
*AEN*	5′- CCCAAATCAGGCGTTCTT	5′- ATAGTCCTTGTGGGCAATTC	XM_003121858.4
*PLK1*	5′- GGTTTCCGCTCAAACATCTA	5′- GCAACTCTATGCCTACATACC	XM_021086465.1
*YWHAG*	5′- TCCCATCACTGAGGAAAACTGCTAA	5′- TTTTTCCAACTCCGTGTTTCTCTA	XM_005661962.3

Primer sets with standard curve R^2^values of ≥0.99 and efficiencies of 95%–105% were selected. Efficiency tests for each primer set were conducted by generating a standard curve of 1:10 dilutions from a 50 ng/μL pooled cDNA reference sample. Each biological replicate was used in triplicate for qPCR on the QIAquant 96 5plex system (Qiagen) to determine differential expression of the selected transcripts with the following conditions: 95°C for 3 min, 40 cycles of 95°C for 10 s, 55°C for 10 s, and 72°C for 30 s. A dissociation curve was generated after amplification to ensure a single product was amplified.

Abundance of each mRNA transcript was calculated relative to a reference cDNA sample and the 14-3-3 gamma protein (*YWHAG*) housekeeping gene as previously described [9]. The pooled cDNA reference was made from four biological replicates of embryos fertilized and cultured in vitro*.*To determine relative mRNA expression for each treatment, the 2^-ΔΔCt^method was used.

### Embryo transfer

To determine if culture with PFT-α had an effect on developmental competence, Day 6 blastocyst stage embryos that had been cultured in MU4 with 50 μM of PFT-α were placed in 3 mL of manipulation medium (9.50 g TCM-199, 0.05 g NaHCO_3_, 0.75 g HEPES, 1.76 g NaCl, 3.00 g BSA, 1 mL gentamicin, 1000 mL Milli Q H_2_O) with 5 μM 5-(4-Chloro-phenyl)-3-phenyl-pent-enoic acid (PS48) in polystyrene tubes (BD Biosciences, San Jose, CA). After transport to the University of Missouri Swine Research Complex at 37°C, 54 blastocysts were loaded into a tomcat catheter and surgically transferred into the ampullary-isthmic junction of a cycling gilt on Day 3 of her estrous cycle. Ultrasound monitoring was conducted after Day 25 to confirm pregnancy status. Surrogates were checked weekly until collection of fetuses or farrowing. Sexes and birth weights were recorded.

### Statistical analysis

All data are presented as mean ± standard error of the mean from at least three independent experiments to account for variability between biological replicates. Analyses were performed by using R (version 4.4.1), and data deviating from normality after the Shapiro–Wilk test were log transformed. The data of development to the blastocyst stage, total cell number, and apoptotic indexes were analyzed by using a one-way Analysis of Variance (ANOVA). Type I error rate was controlled at the level of 0.05. Tukey honest significant difference test was used to analyze differences between treatment groups.

## Results

### Transcriptional profiles differ between three sources of porcine embryos

**Figure 1:**
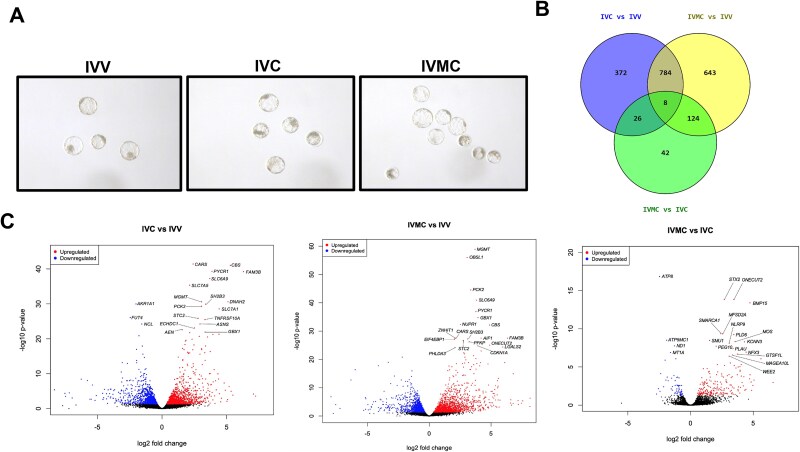
(A) Representative images of the three types of embryos used for RNA sequencing. IVV: in vivo-derived; IVC: in vivo-matured, in vitro-cultured; IVMC: in vitro-matured and cultured. (B) Venn diagrams demonstrating differentially expressed genes (FDR < 0.05 and log2 fold change ≥1) between groups and overlap of the comparisons. (C) Volcano plots demonstrating upregulated and downregulated genes in the first group over second group. Comparison of IVC vs IVV had 1190 differentially expressed genes (489 downregulated and 701 upregulated). Comparison of IVMC vs IVV had 1559 differentially expressed genes (435 downregulated and 1124 upregulated). Comparison of IVMC vs IVC had 200 differentially expressed genes (32 downregulated and 168 upregulated). The top 20 most differentially expressed genes for each comparison are labeled on the plot.

**Figure 2:**
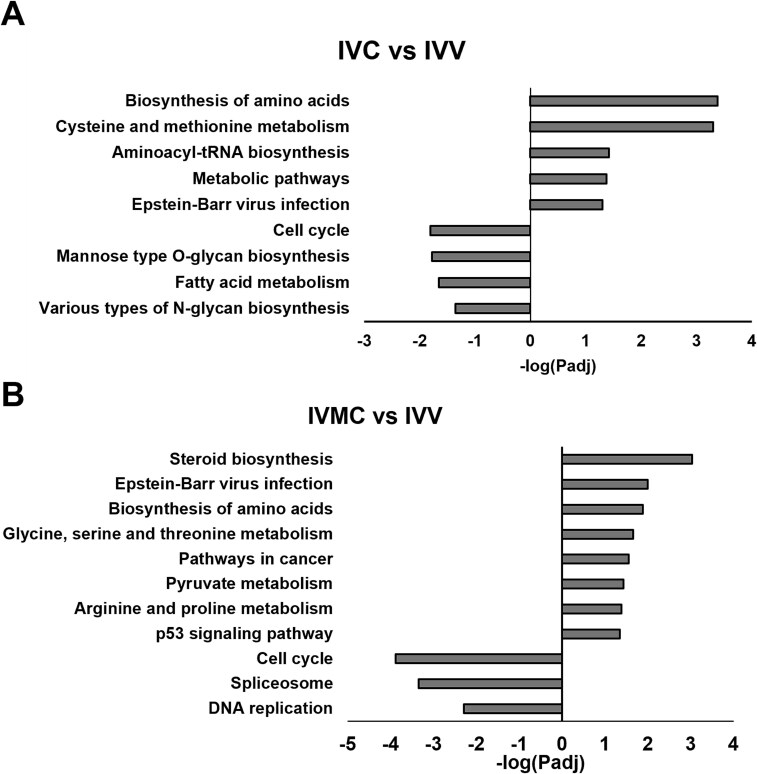
KEGG pathway analysis of three different groups of embryos. (A) Upregulated (positive) and downregulated (negative) pathways in IVC embryos compared to IVV embryos. (B) Upregulated and downregulated pathways in IVMC embryos compared to IVV embryos. Pathway enrichment was not detected between IVC and IVMC embryos.

Transcripts were differentially expressed (FDR < 0.05, log2 fold change ≥1) between IVC vs IVV embryos with 489 downregulated and 701 upregulated, between IVMC vs IVV embryos with 435 downregulated and 1124 upregulated, and between IVMC vs IVC embryos with 32 downregulated and 168 upregulated (Figure 1A and C, Supplementary Table S1). Numerous overlapping differentially expressed genes (DEGs) for the IVC vs IVV and IVMC vs IVV comparisons were observed (Figure 1B). Pathways related to cell cycle were downregulated in IVC and IVMC compared to IVV embryos, and pathways related to amino acid transport in metabolism were upregulated in IVC and IVMC compared to IVV embryos (Figure 2A and B). Of particular interest, the p53 signaling pathway was only increased in IVMC embryos that are entirely produced in vitro compared to IVV embryos (Figure 2B), which may indicate higher levels of stress in the IVMC embryos due to the artificial environment.

### Addition of pifithrins to the medium resulted in different developmental outcomes

**Figure 3:**
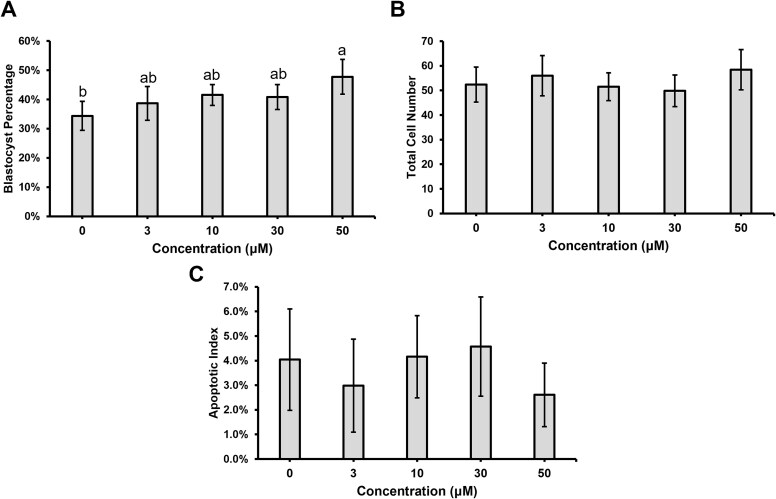
Development to the blastocyst stage after culture with different concentrations of pifithrin-α. (A) Percentage of embryos that developed to the blastocyst stage on Day 6, determined from eight replicates (*n* = 20–60 presumptive zygotes per treatment per replicate). Different letters indicate statistical significance (P < 0.05). (B) Total cell number in Day 6 blastocyst stage embryos, determined from four replicates (*n* = 10 per treatment per replicate). (C) Apoptotic indexes determined by TUNEL staining. Three replicates (*n* = 10 per treatment per replicate). Data are presented as mean ± standard error of the mean.

Different p53 inhibitors, called pifithrins, were added to the porcine embryo culture medium to determine if early developmental arrest could be blocked and development to the blastocyst stage would be enhanced. Pifithrin-α was added to the medium at 0 μM, 3 μM, 10 μM, 30 μM, or 50 μM, which were concentrations used in previous studies [5, 6, 8]. Culturing porcine embryos with 50 μM of PFT-α resulted in an increase in the percentage of embryos that developed to the blastocyst stage compared to 0 μM control embryos cultured only with DMSO (*P* < 0.05; Figure 3A), and higher concentrations (75 μM or 100 μM) did not increase development compared to the control embryos (data not shown). No differences in total cell number or apoptotic index were detected between any of the groups (Figure 3B and C, Supplementary Fig. S4).

**Figure 4:**
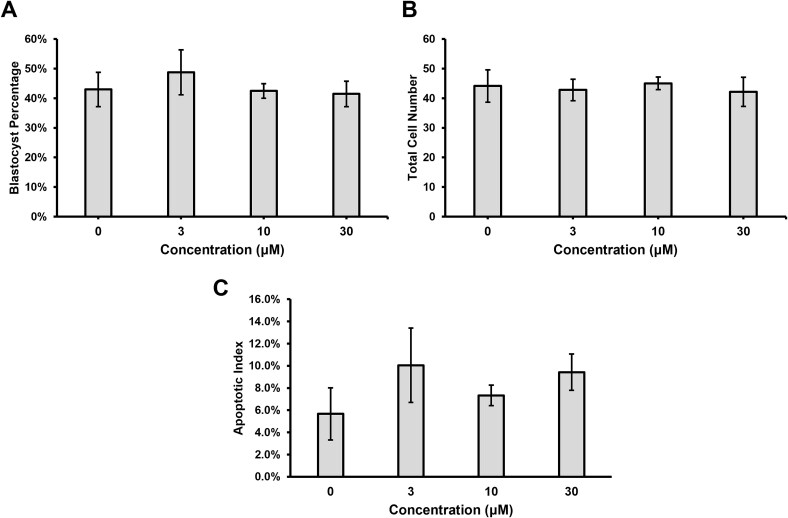
Blastocyst development after culture different concentrations of PFT-β. (A) Percentage of embryos that developed to the blastocyst stage at Day 6, determined from three replicates (*n* = 30 presumptive zygotes per treatment per replicate). (B) Total cell number in Day 6 blastocysts, determined from four replicates (*n* = 5–10 per treatment per replicate). (C) Apoptotic indexes determined by TUNEL staining. Three replicates (*n* = 5–10 per treatment per replicate). Data are presented as mean ± standard error of the mean.

Culturing porcine embryos with 0 μM, 3 μM, 10 μM, or 30 μM PFT-β did not result in any observable differences in development to the blastocyst stage nor total cell numbers (Figure 4A and B). Analysis of DNA damage by TUNEL staining did not reveal any difference in apoptotic index when embryos were cultured at any of the concentrations of PFT-β (Figure 4C).

**Figure 5:**
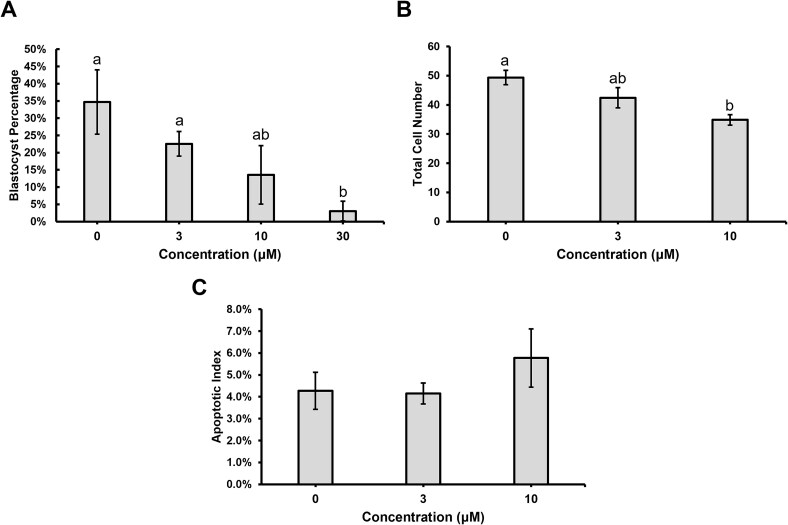
Blastocyst development after culture different concentrations of PFT-μ. (A) Percentage of embryos that developed to the blastocyst stage at Day 6, determined from three replicates (*n* = 30 presumptive zygotes per treatment per replicate). Different letters indicate statistical significance (*P* < 0.05). (B) Total cell number in Day 6 blastocysts, determined from four replicates (*n* = 2–8 per treatment per replicate). (C) Apoptotic indexes determined by TUNEL staining. Three replicates (*n* = 2–8 per treatment per replicate). Data are presented as mean ± standard error of the mean.

Lastly, porcine embryos were cultured with 0 μM, 3 μM, 10 μM, or 30 μM PFT-μ. Culture with 30 μM PFT-μ resulted in a decreased in development to the blastocyst stage compared to embryos with 0 μM or 3 μM PFT-μ (*P* < 0.05; Figure 5A). Total cell number was decreased in embryos cultured with 10 μM PFT-μ (*P* < 0.05; Figure 5B); however, apoptotic index was not observed to be different between the groups (Figure 5C). Insufficient numbers of blastocyst stage embryos for 30 μM PFT-μ were available for assessing total cell number or apoptotic index.

### Pifithrin-α does not affect p53 target gene expression at the blastocyst stage

**Figure 6:**
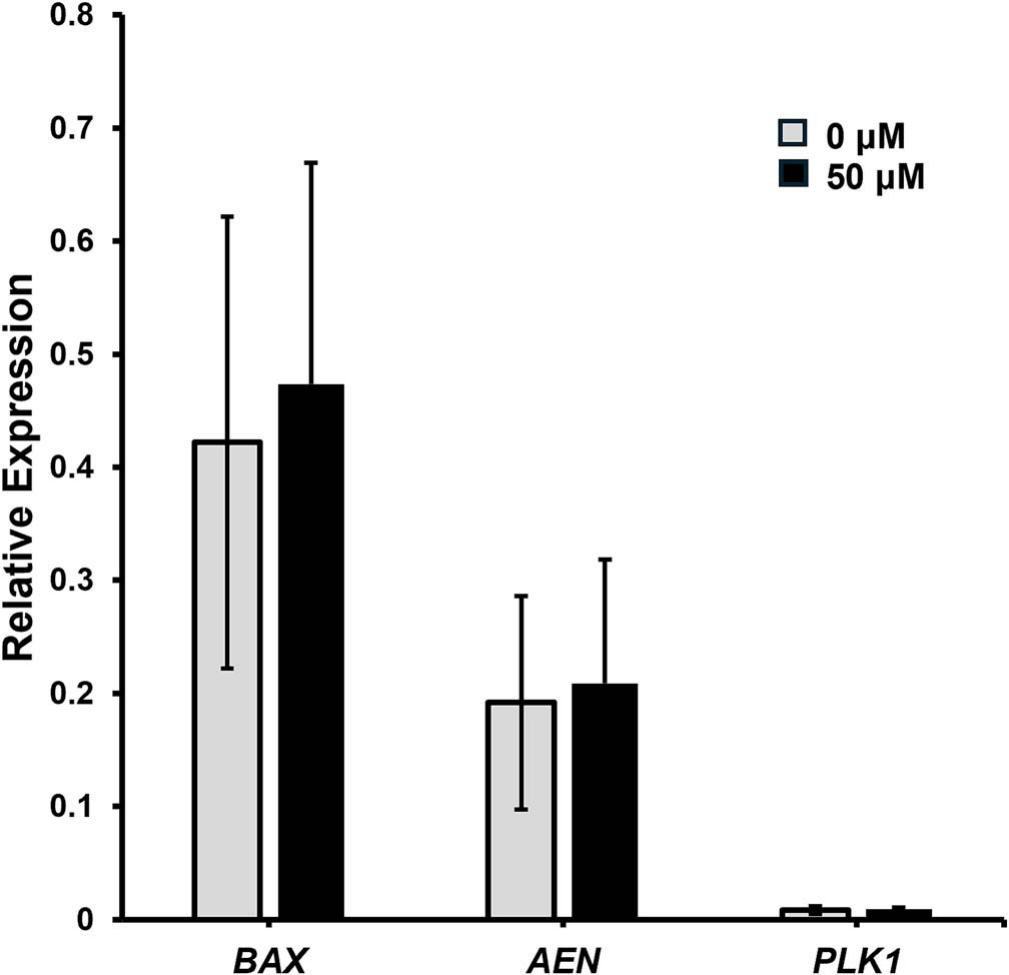
Effects of PFT-α supplementation (50 μM) on relative abundance of target transcripts of p53 in blastocyst stage embryos. Values were determined from 3 replicates (*n* = 30 per treatment). Data are presented as mean ± standard error of the mean.

Quantitative PCR was performed on embryos cultured with 50 μM of PFT-α compared to control embryos. Bcl-2 associated x protein *(*BAX) dimerizes with B-cell leukemia/lymphoma protein 2 (BCL2) to promote apoptosis (Supplementary Table S2). Apoptosis enhancing nuclease (AEN) contributes to apoptotic DNA degradation, and polo-like kinase 1 (PLK1) promotes cell cycle entry (Supplementary Table S2). Despite being direct targets of p53, no difference in transcript abundance was detected for these genes between the control (0 μM) and the PFT-α-treated (50 μM) embryos (Figure 6).

### PFT-α treated embryos are developmentally competent In vivo

To ensure that culture with PFT-α did not abolish developmental competence, two embryo transfers were performed with embryos cultured with 50 μM of PFT-α. Blastocyst stage embryos that had been cultured with 50 μM of PFT-α were transferred into two surrogate gilts (*n* = 54 per gilt), both establishing pregnancies by Day 25. Fetuses were collected on Day 45 from one pregnancy with two males, two females, and one regressing fetus (Supplementary Figure S5). Measurements are presented in Supplementary Table S3. The other gilt farrowed six healthy piglets (three males and three females) on Day 115 of gestation (Table 2).

**Table 2:** Measurements of piglets born after embryo culture with 50 μM PFT-α. G: gilt; B: boar

**Piglet**	**Weight (kg)**	**Sex**
1	0.63	G
2	0.61	G
3	0.54	G
4	0.63	B
5	0.60	B
6	0.62	B

## Discussion

In 2010, Bauer et al. discovered that the transcriptome of porcine embryos cultured in vitro is altered from the in vivo state [1]. These changes are likely induced by the suboptimal, artificial culture environment. The differences in transcript abundance between these two sources of embryos prompted changes to the culture medium to more closely mimic the embryo’s natural environment. After several changes to the culture medium, repetition of RNA-sequencing was necessary; however, three sources of embryos were included in the newest iteration: IVV, IVC, and IVMC porcine blastocyst-stage embryos. As expected, the IVC and IVMC embryos were more similar although DEGs were present. Compared to IVV embryos, IVC and IVMC embryos had upregulation of pathways related to amino acid synthesis and metabolism, indicating that the previous changes to the culture medium may not have drastically impacted the transcriptional profiles of the embryos [2, 3]. One striking difference was that the IVMC embryos demonstrated upregulation of the p53 signaling pathway compared to the IVV embryos while the IVC embryos did not. Therefore, production of porcine embryos entirely in vitro, including both maturation and culture, increases abundance of transcripts related to p53, indicating a state of stress that may lead to early developmental arrest. Inhibition of p53 was investigated as a strategy to overcome the stress response and improve development of porcine embryos to the blastocyst stage.

Low levels of p53 are expressed in normal cells, as p53 is typically flagged for degradation mediated by the RING-finger type E3 ubiquitin protein ligase, mouse double minute 2 homolog (MDM2) [10–12]. If a cell experiences stressors, including heat shock, nutrient deprivation, oxidative stress, or DNA damage, p53 is stabilized. As a result, p53 accumulates in the nucleus, causing degradation of MDM2 and cell cycle arrest or apoptosis [13]. DNA damage can induce phosphorylation of Ser-15, 20, and 46 to activate p53; in addition, phosphorylation at the carboxyl-terminus increases the DNA binding activity of p53 [14, 15]. DNA damage can also induce acetylation of p53 to reduce ubiquitination and increase its stability [16]. Once post-translationally modified and stable, p53 acts as a transcription factor to over one thousand gene targets for cell cycle arrest, DNA damage response, apoptosis, and autophagy.

Higher levels of p53 are expressed by IVC mouse embryos than IVV mouse embryos [17]. Despite transcriptional silence before embryonic genome activation at the 4-cell stage, p53 has been demonstrated to be active in early porcine embryos, which are arresting [18]. Bcl-2 associated x protein accumulation and elevated BAX transcripts in the transcriptional profiling dataset confirm increased activity of p53 in both mouse and pig embryos cultured in vitro [19]. This is likely due to improper composition of the embryo culture medium and handling stress during in vitro production.

Interestingly, p53 is not required for mouse embryos to be developmentally competent as p53 knockout mouse zygotes were more likely to reach the blastocyst stage than wild-type embryos [20]. While both types of embryos were observed to have a similar implantation rate, a larger proportion of the p53 knockout embryos became fetuses than the wild-type embryos. Postnatally, p53 knockout pups appeared normal, but later in adulthood, they had an increased risk for tumorigenesis.

Rather than knocking out p53, an inhibitor can temporarily decrease its activity to overcome the stress response in vitro and allow embryos to reach the blastocyst stage for transfer into a surrogate. A select family of compounds, called pifithrins, has been used to study cancer in cell lines [5]. These molecules can inhibit the transcriptional regulatory activity of p53; however, the exact molecular mechanisms remain unknown. Potential mechanisms of action of pifithrins include inhibiting nuclear translocation of p53 and DNA-binding activity, which is mediated through altered post-translational modifications [6].

The efficacy of pifithrins has been put into question as they do have off-target effects. For example, PFT-α inhibits gene expression differentially, depending on the nature of the target gene, and has also been demonstrated to act as an antioxidant [21]. Other off-target effects of pifithrins include inhibition of other p53 family proteins, such as p73 in zebrafish, p63 in epidermal stem cells, preventing of repression of nuclear factor kappa-light-chain-enhancer of activated B cells activity, blocking activation of alternate receptors involved in initiation of apoptosis, activation of protein kinase B and extracellular signal-regulated kinase, and affecting transcription of p53-independent genes involved with DNA repair, apoptosis, cell cycle, and growth regulation [6, 22–27]. Despite these shortcomings, PFT-α has been widely used to protect cells from DNA damage-induced apoptosis [28]. Pifithrin-β is described as having a 10-fold greater potency and 50% longer half-life than PFT-α. Pifithrin-μ blocks p53 interaction with Bcl-2 and BAX proteins while also preventing its movement into the mitochondria. Bcl-2 and BAX are partnered regulators of programmed cell death [29].

The three pifithrins were individually added to the porcine embryo culture medium to determine their effects on development. The culture of porcine embryos with PFT-β had no observable effect on development, while culture with PFT-μ had a negative impact on development. However, embryos cultured 50 μM PFT-α had increased development to the blastocyst stage on Day 6. Total cell number and apoptotic index were not altered by the presence of PFT-α despite previous studies showing a pro-apoptotic effect of PFT-α on porcine embryos [30]. In the current study, the apoptotic indexes of both control and PFT-α treated embryos were low, which decreases the ability to detect biological differences attributed to p53 inhibition. Thus, culture with PFT-α allowed more embryos to reach the blastocyst stage without compromising embryo quality. Transfers of embryos cultured with PFT-α resulted in healthy fetuses and piglets, which confirmed that the compound is compatible with development in utero. When analyzing transcriptional targets of p53, neither *BAX*, *AEN*, nor *PLK1*transcript abundance differed between control and PFT-α treated embryos. The number of embryos for qPCR analyses was limited for this study; therefore, only three out of hundreds of p53 targets were able to be investigated. Future analyses may include RNA-sequencing of embryos cultured with PFT-α to understand transcriptional changes associated with p53 on a global scale.

Overall, the current study demonstrated that p53 signaling is upregulated in entirely in vitro-produced embryos, and culture with a p53 inhibitor, PFT-α, improves development of porcine embryos to the blastocyst stage and produces embryos capable of establishing pregnancy. Therefore, the addition of PFT-α to the embryo culture medium can be a strategy for increasing the number of blastocyst stage embryos for transfer. Further investigation of the effects of PFT-α on porcine embryos at the molecular level will provide insights into its mechanism of action.

## Supplementary Material

Suppl_Table_S1_(1)_ioaf113

Suppl_Table_S2_(1)_ioaf113

Suppl_Table_S3_(1)_ioaf113

Supplementary_Figure_S4_(1)_ioaf113

Supplementary_Figure_S5_(1)_ioaf113

## Data Availability

All data generated during this study are published within this article or available upon request from the corresponding author. The RNA sequencing data is available on the National Center for Biotechnology Information website (NCBI), reference number PRJNA1165891.
