# Supplementary material for: Transcriptional profiling reveals upregulation of p53 signaling in porcine embryos produced in vitro
Source: Biol Reprod. 2025 May 14;113(4):777–86. doi: 10.1093/biolre/ioaf113 (PMC12527240; doi:10.1093/biolre/ioaf113)
Supplement: Suppl_Table_S1_(1)_ioaf113 [file suppl_table_s1_(1)_ioaf113.docx]

| **Supplementary Table S1.** Transcript differences between IVV, IVC, and IVMC embryos. | | | | | | |
| --- | --- | --- | --- | --- | --- | --- |
| **Comparison** | **Total Transcripts** | **Differentially Abundant (FDR<0.05 & log2 FC)** | **Downregulated or Upregulated** | |  |  |
| IVC vs IVV | 11,553 | 1190 | | 489 | |  |
|  |  |  |  | 701 | |  |
| IVMC vs IVV | 11,844 | 1559 | | 435 | |  |
|  |  |  |  | 1124 | |  |
| IVMC vs IVC | 11,818 | 200 | | 32 | |  |
|  |  |  |  | 168 | |  |
