# Supplementary material for: Transcriptional profiling reveals upregulation of p53 signaling in porcine embryos produced in vitro
Source: Biol Reprod. 2025 May 14;113(4):777–86. doi: 10.1093/biolre/ioaf113 (PMC12527240; doi:10.1093/biolre/ioaf113)
Supplement: Suppl_Table_S2_(1)_ioaf113 [file suppl_table_s2_(1)_ioaf113.docx]

**Supplementary Table S2. Target genes of p53.**

| Gene | Function | IVV Reads | IVMC Reads | P-adj |
| --- | --- | --- | --- | --- |
|  |  |  | | |
| p21^WAF1^  (CDKN1A) | Cell cycle arrest & DNA damage repair | 155 | 2364 | 1.72e^-25^ |
| p53R2 (RRM2B) | G2/M arrest& & DNA damage repair | 74 | 249 | 1.71e^-5^ |
| GADD45α | Growth arrest & DNA damage repair w/ PCNA & p21 | 3126 | 5641 | 0.0106 |
| BTG2 | G1/S transition regulation | 123 | 532 | 4.69e^-4^ |
| KITLG | Hematopoiesis, melanogenesis, gametogenesis, proliferation | 1446 | 948 | 0.370 |
| XPC | DNA damage detection & nucleotide excision repair | 85 | 364 | 2.18e^-5^ |
| PCNA | Indirect target through p21.  DNA replication | 10933 | 9269 | 0.311 |
| POLH | DNA replication | 115 | 56 | 0.178 |
| MDM2 | Ubiquitination of p53 | 1480 | 3781 | 2.38e^-6^ |
| BAX | Dimerizes with Bcl-2 to promote apoptosis | 427 | 1293 | 1.65e^-9^ |
| NOXA (PMAIP1) | Promotes apoptosis, inhibition of anti-apoptotic genes | 616 | 693 | 0.709 |
| AEN | Apoptotic DNA degradation | 1531 | 4334 | 9.76e^-17^ |
| DRAM1 | Autophagy induction | 383 | 505 | 0.430 |
| PRKAB1 | Regulatory subunit of AMPK, monitors cellular energy status | 1256 | 1206 | 0.851 |
| AMPK  (PRKAA1) | Blocks mTOR pathway, resulting in autophagy. Indirect target of p53 via PRKAB1 | 631 | 414 | 0.080 |
| PLK1 | Entry into mitosis and cell division | 8420 | 4878 | 2.80e^-6^ |
