# Supplementary material for: Transcriptional profiling reveals upregulation of p53 signaling in porcine embryos produced in vitro
Source: Biol Reprod. 2025 May 14;113(4):777–86. doi: 10.1093/biolre/ioaf113 (PMC12527240; doi:10.1093/biolre/ioaf113)
Supplement: Suppl_Table_S3_(1)_ioaf113 [file suppl_table_s3_(1)_ioaf113.docx]

**Supplementary Table S3.** Fetal measurements of embryos supplemented with 50 μM PFT-α at day 45 of gestation.

| **Fetus** | **Length (cm)** | **Weight (g)** | **Sex** |
| --- | --- | --- | --- |
| 1 | 5.5 | 14.38 | G |
| 2 | 7.0 | 22.48 | B |
| 3 | 6.5 | 21.49 | G |
| 4 | 7.5 | 25.09 | B |
