# Supplementary figures and images for: Transcriptional profiling reveals upregulation of p53 signaling in porcine embryos produced in vitro
Source: Biol Reprod. 2025 May 14;113(4):777–86. doi: 10.1093/biolre/ioaf113 (PMC12527240; doi:10.1093/biolre/ioaf113)

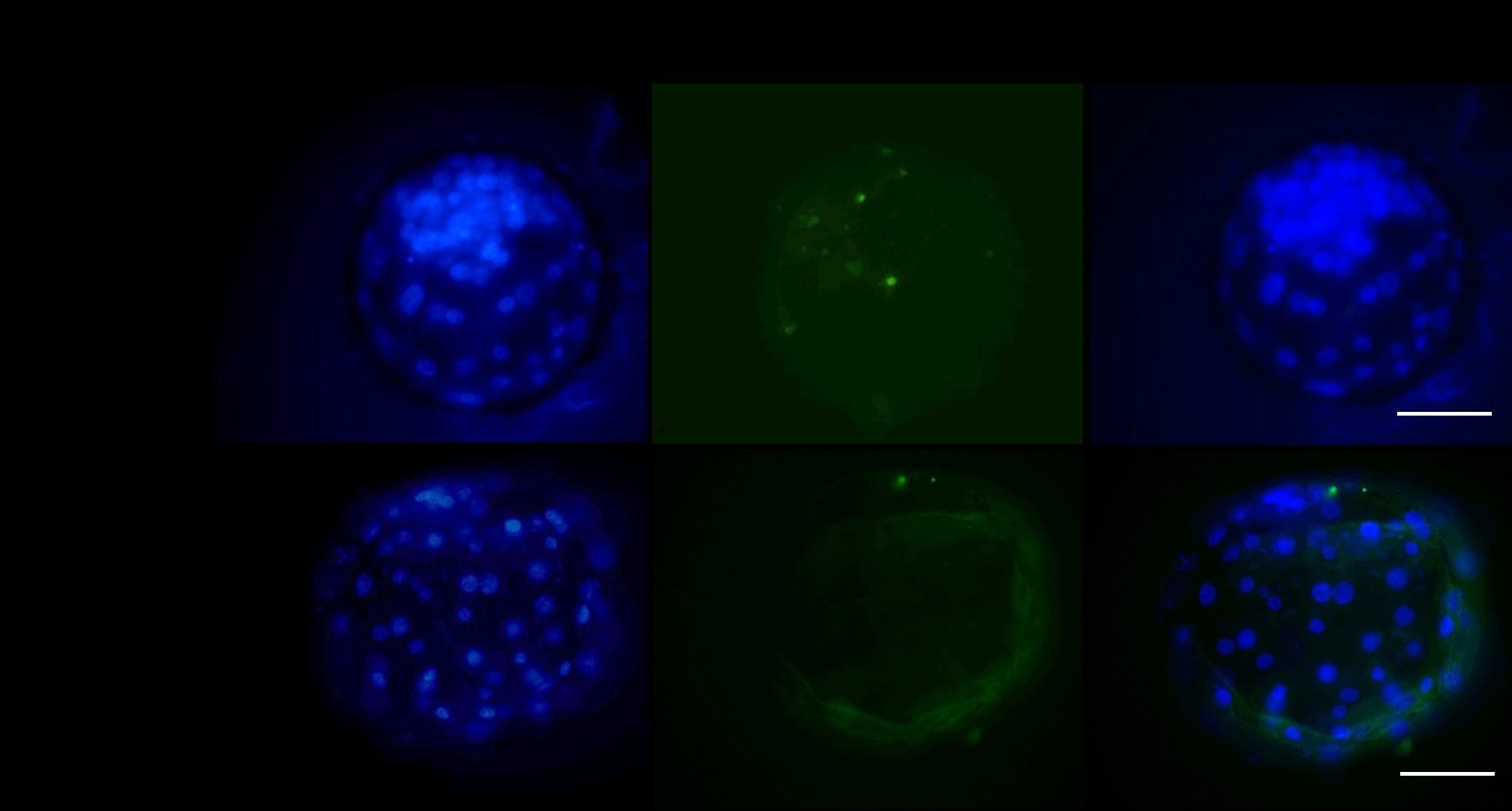

Supplement: Supplementary_Figure_S4_(1)_ioaf113 [file supplementary_figure_s4_(1)_ioaf113.jpeg]

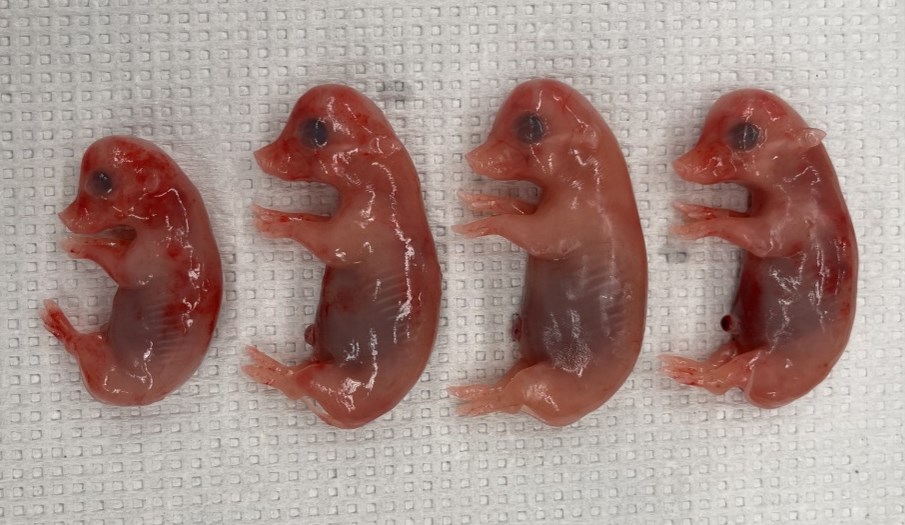

Supplement: Supplementary_Figure_S5_(1)_ioaf113 [file supplementary_figure_s5_(1)_ioaf113.jpeg]
